# Clinical characteristics, long-term complications and health-related quality of life (HRQoL) in children and young adults treated for low-grade astrocytoma in the posterior fossa in childhood

**DOI:** 10.1007/s11060-018-03085-9

**Published:** 2019-01-08

**Authors:** Ingela Kristiansen, Margareta Strinnholm, Bo Strömberg, Per Frisk

**Affiliations:** Department of Women’s and Children’s Health, Uppsala University and Uppsala University Childrens’ Hospital, 75185 Uppsala, Sweden

**Keywords:** Low-grade astrocytoma, Posterior fossa, Childhood, Long-term outcome, Learning difficulties

## Abstract

**Introduction:**

Pilocytic astrocytoma is the most common brain tumour in childhood but knowledge concerning its long-term outcome is sparse. The aim of the study was to investigate if children treated for low-grade pilocytic astrocytoma in the posterior fossa had complications affecting physical and psychological health, cognitive functions, learning difficulties and HRQoL.

**Methods:**

A descriptive single-centre study, where 22 children and young adults out of 27 eligible patients (81%) treated for pilocytic astrocytoma, with a mean follow-up time of 12.4 years (5–19 years) participated (14 adults, two by telephone interviews and eight children). The study included a review of medical records, an interview, neurological investigation, screening tools for psychiatric symptoms (Beck Depression and Anxiety Inventories and Beck Youth Inventory Scales) and HRQoL measures (RAND-36).

**Results:**

Motor complications were most common, reported in 12 patients and mainly affecting fine-motor skills. Seven patients reported cognitive difficulties affecting performance in school. Educational support was given in the period immediately after treatment but not after primary school. None had elevated levels of psychiatric symptoms and the level of HRQoL as well as their psychosocial and educational situation was in correspondence with Swedish norms. The HRQoL score for vitality (VT) almost reached statistical significance.

**Conclusions:**

The long-term functional outcome for children treated for low-grade astrocytoma is favourable. However, some patients report neurological complications and learning difficulties, which are unmet in school. Therefore, there is a need to identify those who need more thorough medical and cognitive follow-up programmes including interventions in school.

## Introduction

The yearly incidence of brain tumours in Sweden during 1984–2005 was 4.2/100,000 in children younger than 15 years [1]. Survival rates have improved but vary across different tumour types [2]. There is also variation regarding long-term complications, including neurological and endocrinological dysfunction, as well as cognitive and psychological difficulties [3, 4]. This may be caused by the tumour itself or by the treatment given (surgery, irradiation and/or cytotoxic drugs) [5]. Complications are mainly reported among children treated for high-grade tumours [6], but in an earlier study we found physical and cognitive complications among children treated not only for high-grade but also for low-grade tumours [7]. The tumour location plays a role in that infratentorial tumours are described to have a worse prognosis compared with supratentorial tumours, especially regarding cognitive and social-emotional functioning [8]. This may be explained by the role of the cerebellum in coordinating motor function and cognition [9]. Studies using functional MRI have shown that the cerebellum coordinates executive functions and working memory in neural networks. Injuries may affect executive function, spatial cognition, personality and language [9] as well as fine-motor skills, most obvious in complex activities [10]. Children treated for tumours in the posterior fossa also perform worse in tests of gross-motor functions, especially when performing balance tasks [11]. A cerebellar cognitive affective syndrome has been described, characterised by deficits in executive function, spatial cognition, linguistic processing and affect regulation [12]. Difficulties are reported for all children treated for tumours in the posterior fossa, but more pronounced in children treated with both surgery and radiation [10].

Pilocytic astrocytoma is the most common brain tumour type in childhood located in the posterior fossa, and is mainly treated with surgery [13, 14]. These tumours have low mortality, and the long-term functional outcome is described as favourable [15–17]. Although there are studies describing neurological, cognitive, emotional and behavioural complications among these patients there is still a lack of knowledge concerning the long-term outcome for this population [18–24].

Against this background of ambiguous results concerning the long-term functional outcome for children treated for low-grade pilocytic astrocytoma, the aim of this descriptive study was to investigate whether patients had long-term complications affecting physical and psychological health, cognitive functions and quality of life. We also wanted to investigate if this affected the psychosocial and educational situation for young adults and whether learning difficulties had been observed in school and had led to increased educational support. Our hypothesis was that some of the patients have long- term complications, affecting physical and psychological health, cognitive functions including learning abilities, and quality of life.

## Materials and methods

### Participants

This single-centre study was performed at Uppsala University Children’s hospital, Sweden, a tertiary referral centre for children with CNS tumours. The centre serves six counties with a population of 1.8 million inhabitants. Patients were retrieved from the local and the National Brain Tumour Registry. A total of 27 patients < 18 years of age with a low grade astrocytoma in the posterior fossa were diagnosed and treated in childhood between 1995 and 2011. At the time of this investigation, nine were children (9–17 years) and 18 young adults (21–33 years). Hospital medical records were retrieved from paediatric, neuropaediatric, neurooncology and neurosurgery departments as well as neuropsychology records, including pre- and postoperative assessments. The original neuropathological slides for patients diagnosed between 1995 and 2006 were re-evaluated by two neuropathologists [7], and the tumours were classified according to the World Health organisation (WHO) Classification of Tumours of the Central Nervous System [7, 13]. For this study, no further re-evaluation was performed for those diagnosed 2007–2011 (n = 7). However, no changes from the original neuropathological diagnoses were seen in the re-evaluated tumours from 1995 to 2006.

**Fig. 1:**
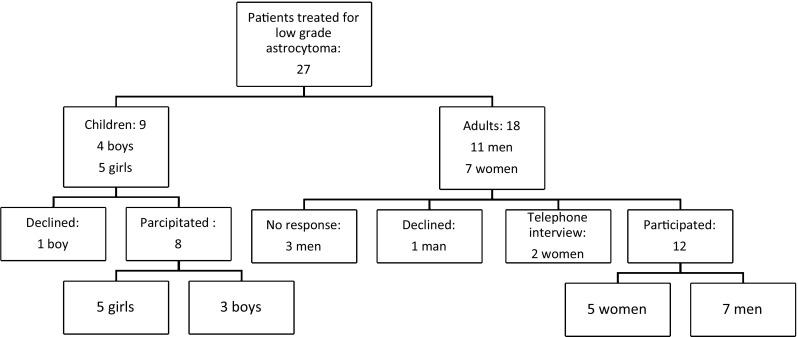
Participants in the study

A total of 22 out of 27 eligible patients agreed to participate (81%), 14 adults and 8 children (Fig. 1). Of these, two adults only took part in telephone interviews; one of them was excluded for further participation due to an intercurrent neurological condition (multiple sclerosis), and the other declined to participate. Three patients did not answer several invitations to participate in the study, and 2 patients declined to participate.

### Methods

Upon acceptance, participants were asked to come to the Folke Bernadotte Regional Rehabilitation Unit to undergo an interview and a neurological investigation by an experienced paediatric neurologist, (IK). Adult participants, including one who took part only in the telephone interview, completed a form for health-related quality of life, RAND-36.

Children and adults completed screening tools for psychiatric symptoms (Beck Youth Inventory Scales, Beck Depression Inventory, second edition, and Beck Anxiety Inventory). All tools are validated and available in Swedish.

#### HRQoL

##### RAND-36

RAND-36 is one of the most commonly used generic measures for HRQoL, developed in the RAND Medical Outcome Study during the 1980s. Two identical versions of the questionnaire are currently available [25]. The RAND-36 Item health survey, is a public domain form, and the SF-36 Item health survey is a copyrighted, commercially distributed form. Minor differences exist between RAND-36 and SF-36 concerning scoring procedures for two of the eight subscales. RAND-36 lacks an authorised algorithm for calculating mental and physical summary scores. Both RAND-36 and SF-36 are available in Swedish [25]. The questionnaire consists of 36 items measuring eight HRQoL domains: physical functioning (PF), role physical (RP), bodily pain (BP), general health (GH), vitality (VT), social functioning (SF), role emotional (RE), and mental health (MH). Domain scale scores range from 0 (worst possible quality of life) to 100 (best possible quality of life). Swedish age- and gender-matched norm values exist for domain scores for SF-36 [26]. Due to the identical construction of the questionnaires, it is possible to compare the domain scores from RAND-36 with the Swedish norm values for SF-36.

#### Depression, anxiety and self-esteem measures

##### Beck depression inventory—second edition (BDI–II)

BDI–II is a self-report scale, intended to measure the amount of depressive symptoms in adults and adolescents. The items correspond with criteria for depression in the Diagnostic and Statistical Manual of Mental Disorders—fourth edition (DSM-IV; 1994). The inventory consists of 21 groups of symptoms and attitudes scored from 0 to 3 in severity. BDI–II evaluates both physiological and cognitive depressive symptoms. The results are interpreted in relation to established limit values [27].

##### Beck anxiety inventory (BAI)

BAI is a self-report scale intended to measure amounts and changes in level of anxiety. The inventory consists of 21 statements scored from 0 to 3 in severity. BAI evaluates both physiological and cognitive anxiety symptoms. The results are interpreted in relation to established limit values [28].

##### Beck Youth Inventory Scales (BYI)

BYIs evaluate emotional and social impairments in children and adolescents. Each scale consists of 20 statements, scored 0–3, indicating the frequency of symptoms during the last 1–2 weeks. The scales include: anxiety (BAI-Y), depression (BDI-Y), anger (BANI-Y), disruptive behaviour (BDBI-Y) and self-esteem (BSCI-Y). Scores are reported as percentiles in comparison with Swedish normative data [29].

### Cognitive investigations

Pre- and postoperative neuropsychological investigations were performed by neuropsychologists, using standardised cognitive tests; in most cases WISC (Wechsler Intelligence Scale for Children) [30].

### Statistics

Statistics were calculated using the SPSS statistical program, version 24. Non-parametric tests were used due to the small sample sizes and non-normal distribution of data. One-sample Wilcoxon signed-rank test was used to compare values for RAND-36 with Swedish reference from SF-36. The level of significance was set at p < 0.05.

## Results

### Age distribution, heredity, medical history and mortality

**Fig. 2:**
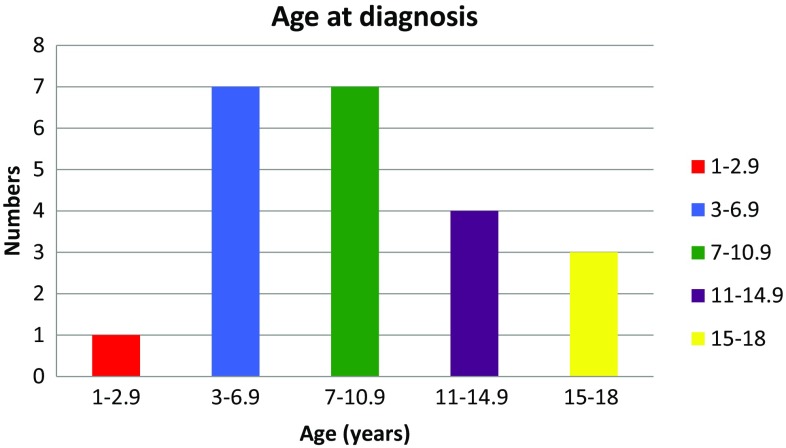
Age at diagnosis

The mean age at diagnosis was 8.7 years (median 8.2 years) with the distribution presented in Fig. 2.

Eighteen patients had no family history of malignant diseases including brain tumours; 3 reported tumours in first-grade relatives (2 cases of breast cancer and 1 lung cancer). Information was unavailable for 1 patient. None of the patients had Neurofibomatosis type 1.

Twenty-one patients evinced normal psychomotor development prior to diagnosis, and 1 patient, diagnosed at 1 year of age had early symptoms from the tumour affecting development of motor function.

No patient had a history of neuropsychiatric disorders. Twenty patients were reported as healthy before the tumour diagnosis. One patient was treated for strabismus and one for leg-length discrepancy due to an infection at an early age. There were no deaths.

The mean age at participation in the study was 20.8 years (median 23.0 years). The youngest patient was 9 and the oldest 33 years. Mean time from diagnosis to the study was 12.4 years (median 12.5 years, range 5–19 years).

### Symptoms at diagnosis and symptom duration

**Fig. 3:**
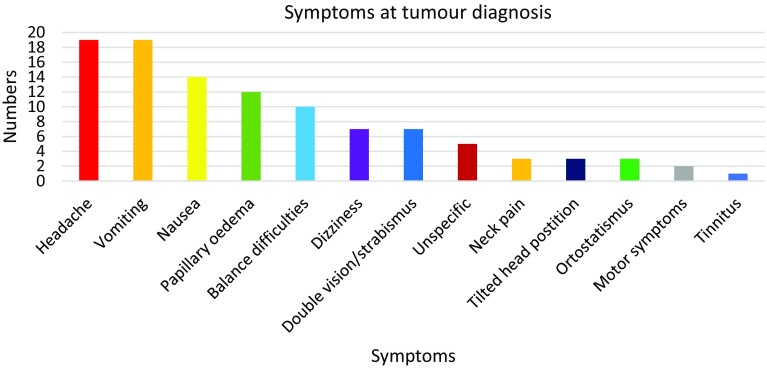
Symptoms at tumour diagnosis

The most common symptoms were headache, vomiting and nausea (Fig. 3). Twelve patients had papillary oedema but information about this sign was lacking in 3 patients. Seventeen patients had hydrocephalus at diagnosis, but no-one needed a ventriculo-peritoneal shunt after surgery.

Twelve patients had symptom duration shorter than 3 months and four patients longer than 12 months.

### Treatment

All patients were treated surgically; in 19 patients the operation was considered radical. Three patients had a remaining tumour and were re-operated shortly after the initial operation. After surgery, 3 patients had CSF leakage and 4 developed meningitis. Three patients relapsed; 1 was treated with surgery, 1 with surgery and chemotherapy and 1 with gamma knife radio surgery. One of these patients had another relapse in October 2018. The final histopathological diagnosis is not yet established, but the tumour seems to have become malignant. The other two patients are still being followed up with MRI investigations.

### Medical outcome

**Fig. 4:**
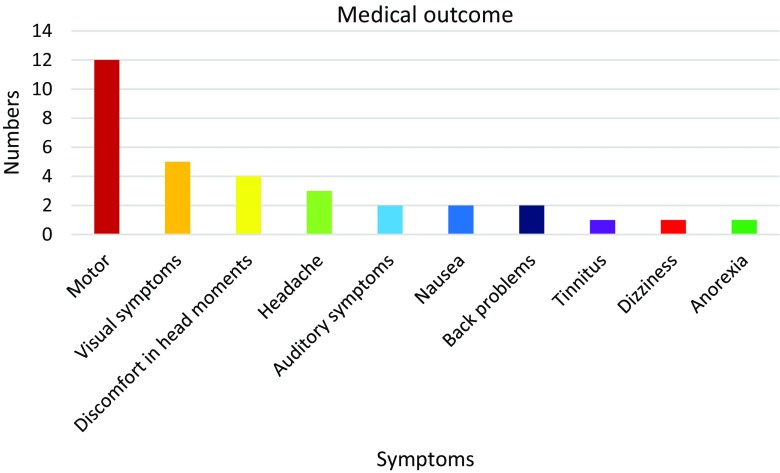
Medical outcome

Motor complications were the most commonly reported sequelae in 12 patients (Fig. 4), mainly influencing fine-motor skills, including tremor, dexterity and reduced strength in the hands. Two patients had changed handedness from right to left. Three patients evinced difficulties with balance. Five patients had visual symptoms, mainly strabismus. One had papillary oedema affecting vision at presentation and persistent visual impairment. Two patients had hearing loss confirmed with audiometry, one after treatment with chemotherapy and the other had unilateral hearing loss already at presentation.

None suffered from posterior fossa syndrome. Patients with a symptom duration > 12 months at presentation, preoperative hydrocephalus and/or postoperative meningitis did not have a worse outcome.

### Psychiatric and neuropsychiatric outcome

Two adults have been treated with anti-depressive medications. No one fulfilled the criteria for a neuropsychiatric diagnosis or has had behavioural difficulties.

### Cognitive outcome

#### Preoperative neuropsychological investigations

Five patients had performed a neuropsychological investigation before tumour surgery. Four had results within average for age and 1 displayed a short auditory attention span.

#### Postoperative neuropsychological investigations

Fifteen patients performed a postoperative neuropsychological investigation; nine had results average for age and six below average för age. There was great variation in the timing of the investigation related to time of surgery; 12 patients performed the first investigations 1 week–15 months postoperatively and three investigations were performed 2, 5 and 8 years after surgery, respectively. There were also variations in the test batteries used. Difficulties included slow processing speed, working memory and auditory processing.

#### Later cognitive outcome and educational performance

In the interviews, 7 patients (3 children and 4 adults) reported cognitive difficulties affecting performance in school and daily life.

In primary school, 11 patients had received extra educational support. No one received extra support in high school (n = 14).

Five adult patients had completed or were studying at university. One patient had studied a few university courses; 3 patients had finished vocational training; and 1 patient took part in adult education. Four patients had not studied after high school.

### Psychosocial outcome

All 8 children lived with their parents. Among the adults, 4 were single; 4 had girl- or boy-friends but did not cohabitate; 1 was single with children; and 5 were married or cohabitated with children. No one reported difficulties with social relations.

### HRQoL

Thirteen patients completed RAND-36. The mean domain scores were comparable with Swedish norms. The score for VT (vitality), was on the level of statistical significance (p = 0.054) (Table 1).

**Table 1:** Results for RAND-36 compared with Swedish norms for SF-36

Domain	Results for participants mean values	SF-36 reference sample; 15–29 years mean values	p values
PF physical functioning	92.69	94.9	0.501
RP role physical	88.46	90.7	0.124
BP bodily pain	80.96	81.2	0.972
GH general Health	72.69	82.0	0.152
VT vitality	63.46	70.8	0.054
SF social function	89.42	91.7	0.803
RE role emotional	84.61	88.2	0.971
MH mental health	79.38	81.8	0.752

### Depression, anxiety and self-esteem

#### Beck anxiety inventory and beck depression inventory

Eleven adults and 1 adolescent aged 17 years completed the inventories; 3 adults did not participate. Ten patients had minimal, 1 had mild and 1 had moderate levels of anxiety. For depression, 11 patients had minimal levels and 1 mild levels of depression. None indicated suicidal thoughts or plans.

#### Beck youth inventories

Seven children completed the questions for the 5 scales. In the scales scoring anxiety, depression, disruptive behaviour and anger all scored average results. For self-esteem, 1 child scored high and the rest average results. None indicated suicidal thoughts.

## Discussion

Pilocytic astrocytoma is the most common brain tumour in childhood and the one with best outcome [14, 15]. However, there are lack of knowledge and ambiguous results concerning long- term outcome. In this study we found favourable long-term outcome with excellent tumour control (survival rate 100% and only three patients relapsed after initial surgery including one with an additional relapse) and favourable functional outcome. Some of the patients showed physical and/or learning difficulties (reported in the interviews) affecting daily living to some extent. This is in accordance with results from other studies showing age-appropriate ability to perform daily activities. The participants in these studies had motor complications (fine-motor skills and balance), difficulties in school and behavioural and emotional adjustment disturbances [15, 17, 23]. Ghazwani et al. [31] reported hearing-loss following surgery, but we were unable to replicate these findings. In a study by Sadighi et al. [32] a prediagnostic symptom interval > 3 months affected some functionally important neurologic outcomes, but in our study we could not find this. Neither did preoperative hydrocephalus or postoperative meningitis affect outcome. The levels of depression and anxiety were not increased among the adults according to results of BAI and BDI–II, though 2 patients had previously been treated with anti-depressive medications. Among the children, all had average scores for all items in Beck Youth Inventory, indicating no increased levels of psychiatric symptoms. In a study by Upadhyaya et al. [33] four cases of suicide were reported. In our sample we found no indications for increased suicidal risk in the questionnaires. Neither were there any difficulties with behaviour or social relations. In a study by Zuzak et al. [17], HRQoL was similar to that of healthy controls. We found the same result in the adult group, but the vitality score was on the level of statistical significance. This score reflects the person’s level of subjective well-being and may indicate an impact of having suffered from a potentially life-threatening disease during childhood. The potential risk for psychological dysfunction in children with low grade glioma, as noted in the study by Upadhyaya [33], underscores the importance of early recognition of psychological symptoms and interventions.

The adult patients showed no signs of psychosocial difficulties and had an educational situation comparable to the general population in Sweden [34], which is also reported in other studies [15, 23]. However, in a study by Pletschko et al. [35] patients treated for cerebellar pilocytic astrocytoma showed satisfactory academic achievement, but when they were compared with high achievers (i e medical students) specific cognitive impairments became apparent. This underscores our findings in the interviews, that as much as one-third of the patients reported learning difficulties that were not dealt with in school, despite the fact that some of these difficulties had been diagnosed in neuropsychological investigations. These patients also reported that they had struggled hard to complete their school work. The extra support given was mainly in primary school and during the time immediately after surgery. No one received extra support in high school.

Thus, despite the favourable prognosis, some of the children and adults treated for a low-grade astrocytoma have complications affecting neurology, cognition and learning. In the sample investigated, these difficulties were not so pronounced that they affected the person’s ability to live a healthy and rich life with work and well-functioning personal relationships. However, for some, the learning difficulties were on such a high level that further support would have been warranted. Together with the reported motor difficulties, this poses an extra burden for these patients.

The limitations of this study comprise the small number of patients and the partly retrospective design, with a varying degree of information available in medical records, especially for neuropsychological data.

The strength of the study is that it was possible to identify all 27 children and young adults diagnosed with low-grade astrocytoma in the posterior fossa during childhood treated in the same tertiary referral centre in Sweden during a defined time period and to follow-up 22 (81%) study participants, which entails a low risk for selection bias. The Swedish registers containing information on children, adolescents and young adults treated for this tumour type were scrutinized; hence we believe that all patients treated in the health region are included. A thorough review of all the medical records has been undertaken in all patients, and we have been able to get broad information about the disease process and later complications. We also have a long follow-up period (mean time 12.4 years; range 5–19 years). Moreover, the fact that we have had personal contact with the patients has given us a deeper understanding of the impact of having been treated for a brain tumour in childhood. This has also given us information that would have been hard to obtain if only information from questionnaires and medical records was used.

## Conclusions

The long-term functional outcome for children treated for low-grade astrocytoma is favourable. However, some patients report neurological complications and learning difficulties which are unmet in school. Therefore, it is important to identify those in need of more thorough medical and cognitive follow-up programmes including interventions in school. Thus, more studies are needed to identify risk factors for both neurological complications and learning difficulties and to develop appropriate intervention programmes. We plan to undertake further investigations concerning motor functions, cognition, language and educational performance and to present these in another paper.
